# Burn Patient Perspectives on Disability Weights and the Philosophy of Disability: A Gap in the Literature

**DOI:** 10.3390/ebj4040037

**Published:** 2023-11-09

**Authors:** Paul Won, Karel-Bart Celie, Cindy Rutter, T. Justin Gillenwater, Haig A. Yenikomshian

**Affiliations:** 1 Keck School of Medicine, University of Southern California, Los Angeles, CA 90033, USA; paulwon@usc.edu; 2 Uehiro Center for Practical Ethics, University of Oxford, Oxford OX1 1PT, UK; karel-bart.celie@med.usc.edu; 3 Division of Plastic and Reconstructive Surgery, University of Southern California, Los Angeles, CA 90033, USA; justin.gillenwater@med.usc.edu; 4 Independent Researcher, Los Angeles, CA 90033, USA; cindyerutter@gmail.com

**Keywords:** disability weights, burns, patient perspective

## Abstract

Background: Disability-adjusted life years (DALY) have a ubiquitous presence in academic global health, including attempts to understand the global burden of burn injuries. Objective: The present scoping review aimed to examine whether disability weights (DWs) were informed by burn patient perspectives and secondarily to determine whether literature indicates which of the three most common philosophical models of disability best aligns with burn patient experiences. Methods: A review of six databases was conducted and The Critical Appraisal Skills Program (CASP) checklist was utilized. Results: Out of a total of 764 articles, zero studies solicited patient perspectives of DWs. Four articles contained data that could be extrapolated to patient perspectives on disability. All articles utilized semi-structured interviews of burn survivors and reported thematic elements including return to work, self-image, and social integration. Patients reported similar themes that burn injuries were disabling injuries and instrumentally detrimental, with modulation based on the patient’s social circumstances. Conclusions: This scoping review highlights a significant gap in literature. First, no studies were found directly investigating burn patient perspectives on burn DWs. Current DWs have been derived from expert opinions with limited input from patients. Second, the limited primary patient data gleaned from this review suggest patients consider their injuries as instrumentally detrimental, which aligns most closely with the welfarist view of disability. More explicit investigations into the philosophical model of disability best aligning with burn patient experiences are needed to ground the health economics of burns in sound theory.

## 1. Introduction

Globally, the World Health Organization (WHO) estimates there are 11 million burn injuries annually, of which over 180,000 are fatal [1]. Burn-injured people often face significant physical and psychosocial challenges, which have significant impacts on their quality of life [2,3,4]. For example, those with burn injuries often face barriers to return to work and social integration due to their burn scars, contractures, and perceived self-image [5]. For the past several decades, studies have sought to better understand the global and individual impact of adverse outcomes following traumatic injuries such as burns [6,7,8]. The lasting, negative impact of burns on patients can be understood as “disability”, though as we will discuss below a precise definition cannot be given without adopting a philosophical account of why or why not something counts as a disability.

The disability-adjusted life years (DALY) model was first employed by the Global Burden of Disease (GBD) group as a way to measure the burden of diseases such as burns (Table 1) [9]. Previously, studies have utilized disability weights for burn conditions by incorporating WHO definitions and data sources to measure disability weights and define health states (Table 1) through empirically derived EuroQol 5 Dimensions (EQ-5D) or Medical Outcomes Short Study Form (SF-36) scales [10]. Generally, the DALY model utilizes both “time lived with a disability” and “time lost due to premature mortality” to assess burden of a particular disease state [9]. The DALY formula is as follows:

$$DALY=Years\;Lived\;with\;Disease\left(YLD\right)+Years\;of\;Life\;Lost\;(YLL)$$

where

YLD=Prevalence×Disability Weight

Note that the disability weight (DW) is a very significant component of the overall formula. It is an assigned number between 0 (state similar to full health) and 1 (state similar to death), indicating the severity of living with a disease state. This number therefore greatly depends on the sources that are queried for its development.

To standardize and better understand the impact of DW, the DALY was intended to help policymakers and stakeholders prioritize resource allocation and health interventions. Since its inception, it has been utilized in a variety of settings; from measuring population health to calculating the cost-effectiveness of public health interventions [11,12,13]. Of note, the DALY was designed to be a measure of burden “based on explicit and transparent value choices” [14]. Therefore, the developers of the DALY initially acknowledged the value-laden nature of the measure. However, in recent years, the DALY has undergone changes aimed at making it purely descriptive (i.e., stripped of value assumptions) [15]. This may explain why normative assumptions (i.e., hypotheses and statements relating to an evaluative standard) underlying the theoretical framework of the DALY have received relatively little attention. This gives rise to two issues.

The first problem relates to how DWs are determined. If the purpose of the DALY is to measure the individual burden of disease—and not simply track disease—we would expect most data collected on DW to come from persons living with the disease states in question (i.e., burn patients). However, this is far from the truth. A 2022 review of DW measurement studies found that, out of 46 studies, only 4 studies (8.7%) included patient groups. In contrast, a staggering 59% included panels of health experts, with the remainder surveying the public or using model estimation methods [16]. This same study reported significant differences between the value judgments of patient and non-patient populations, further emphasizing the importance of incorporating patient values in measures like the DALY. However, as it has been shown that DW valuations of medical experts and the general population differ, with incorporation of the general public into value judgements, valid health state valuation judgements are more difficult to obtain [17]. This is hypothesized to be due to reduced knowledge and experience in the general population regarding the studied outcomes of health states, such as burn injuries [16].

A second issue is the limited understanding of what disability means to burn patients and which philosophical model best underpins theoretical frameworks such as the DALY. This matters because the value assumptions that go into defining disability end up determining what does or does not count as disability, which in turn impacts who might have claims to resources. For example, by far the dominant view in healthcare is the ‘medical model.’ This view defines disability as a stable property that deviates from the scientific or biological truths for the species [18]. An illustrative case of when this may be problematic is hearing loss in old age. If we accept that eventual hearing loss is normal for our species, it becomes more difficult to justify spending healthcare resources on hearing aids strictly based on our definition of disability. In contrast to the ’medical model’, the ‘social model’ of disability does not regard deviations from the species norm as disabling, rather as a ‘mere difference’, which becomes disadvantageous solely due to societal prejudice (Table 2) [19]. However, this model runs into difficulty distinguishing between disability and discrimination. As an alternative, the ‘welfare model’ incorporates ‘the insights of the medical and social models’ while avoiding some of their respective inconsistencies [20]. A key feature of this model is that it assigns intrinsic value to well-being—disabling causes (e.g., blindness) are only instrumentally bad insofar as they reduce well-being.

The three most employed philosophical models of disability are therefore the ‘medical model,’ the ‘social model,’ and the ‘welfarist model’ [20]. Some other models, such as the Nagi Model or the Verbrugge and Jette, have been proposed to better define the experience of disability regarding physical problems and functional limitations. A full description and discussion of the drawbacks and benefits of each model is beyond the scope of this paper, although well described in the literature [21]. However, we want to point out that the value of examining this (in the context of burn patients) is because what we decide does or does not count as disability affects everything else down the line. It is not a matter of if we make value assumptions when discussing concepts like disability and burden. It is a matter of which assumptions we are making. Those assumptions should be more explicitly discussed.

The aim of this scoping review was to evaluate the burn literature to determine whether (1) any studies exist investigating burn patient perspectives for the development of DWs and (2) if any studies indicate which philosophical model of disability corresponds most with burn-injured peoples’ viewpoints.

## 2. Methods

Our Institutional Review Board (IRB) approval was waived due to study design. The study was registered on Open Science Framework (OSF) on 20 March 2023 (registration: https://doi.org/10.17605/OSF.IO/78HEC) to reduce potential for bias and duplicate reviews [23]. Standard guidelines from Preferred Reporting Items for Systematic Reviews and Meta-Analysis for Scoping Reviews (PRISMA-ScR) were utilized [24]. A systematic review of literature using six databases, PubMed, Embase, CINAHL (Cumulative Index to Nursing and Allied Health Literature), Web of Science, PsycInfo, and PhilPapers was conducted for articles published anytime between the earliest possible search time frame and 1 January 2023.

Two authors performed literature review and study assessment for inclusion. To collect articles pertaining to DALY and burn injury, our Boolean search string was ((Burn OR burn patient) AND disability AND (perspective OR viewpoint OR perception OR impression OR point of view)). Inclusion criteria were studies in English, studies with human subjects, and studies investigating burn patient perspectives on disability. Exclusion criteria were case reports, literature reviews, editorials, and position pieces. Articles that met inclusion criteria underwent full text review. Study aims, methodology, and results were collected, and general themes were identified and described. The Critical Appraisal Skills Program (CASP) checklist was utilized to evaluate included studies [25]. This CASP checklist, developed at Oxford University in 1993, is a well-investigated tool comprised of ten questions that provide quality appraisal of qualitative evidence synthesis [26]. The checklist consists of screening questions that assess methodology, results, and organization of the study in question.

## 3. Results

**Figure 1:**
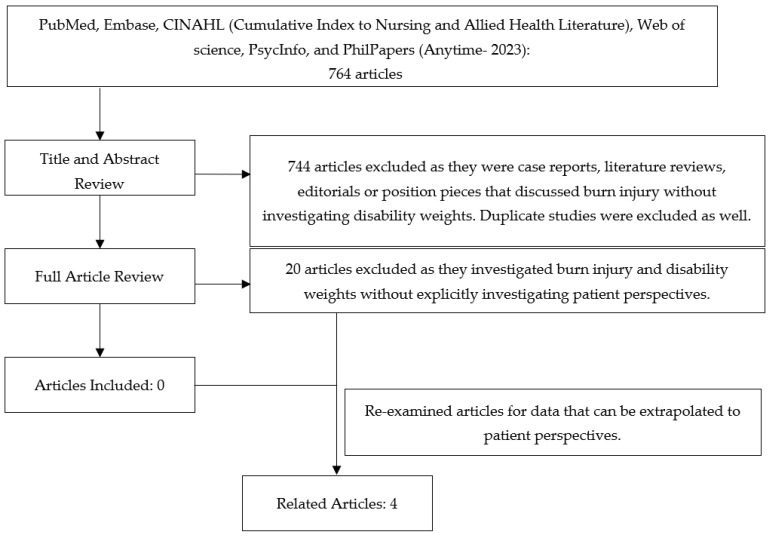
Preferred Reporting Items for Systematic Reviews and Meta-Analysis (PRISMA).

The initial search returned a total of 764 articles. Table 3 displays the total number of articles from each database. After an abstract review and duplicate removal, 744 articles were excluded, with 20 articles remaining for review. After a full text review, no studies were found directly soliciting burn patient perspectives for the development of DWs (Figure 1).

Four articles reported data that could be extrapolated to patient perspectives on the philosophical models of disability. 

Table 4 provides a summary of each of the four included articles, which were published from 2009 to 2014. Two studies were conducted through the WHO Collaborating Center for Nursing Research Development in Brazil [27,28]. One study was based in South Africa, and another in Texas, United States [29,30]. Three articles utilized semi-structured interviews of burn survivors to gather data [27,28,30]. Of these articles, only one, Dunpath et al., utilized the International Classification of Function, Disability, and Health (ICF) framework to develop a qualitative study design [30]. Briefly, the International Classification of Functioning, Disability and Health (ICF) is a conceptual framework based on a biopsychosocial view developed by the WHO in 2001 [31]. This framework provides different outcome categories to guide research investigating patient perspectives on outcomes. Studies employ the ICF to evaluate patient perspectives for conditions, such as hearing loss, Alzheimer disease, and traumatic injuries [32,33,34]. Russell et al. utilized two scales, the Tennessee Self-Concept Scale and the Young Adult Self-Report, and a structured interview that was conducted to all participants [29]. Overall, all articles addressed each element from the CASP Checklist for Qualitative research.

All studies investigated burn patient perspectives on themes related to disability and burden of disease. However, difficulty was noted in gathering these perspectives. For example, Rossi et al. reports that ‘although participants were asked to talk about the meaning of quality of life from their perspective, their answers were mainly focused on the factors associated with good or bad quality of life’ [28]. In other words, patients tended to focus on factors they thought were instrumentally related to their quality of life, such as physical functionality and body image. Although queried, patients were often not able to clearly communicate whether these instrumental outcomes contributed to their perspective of disability, and if so to what degree. Thus, although the current investigation in the literature helps define potential factors to address clinically that would improve a patient’s perceived quality of life, there remains a paucity regarding granularity and detail expanding the burn patient’s perspectives on disability and burden of disease.

Furthermore, themes in all studies included physical, social, and emotional measures such as physical function, social dilemmas, and support systems [27,28,29,30]. Regarding studies investigating burn patients from low-resource communities, themes involving patients’ social environment strongly influenced burn patient perspectives of injury and disability [30]. For example, quality of life was greatly associated with concepts related to autonomy such as return to work or pre-injury activities [28]. Patients with manual occupations often reported a significant burden of disease and cited resumption of work as a significant factor to their social adjustment and burn recovery [27,28]. Patients in all studies reported that body image disturbances significantly affected their quality of life, impacted their self-esteem, and that negative interactions with other people significantly contributed to psychosocial wellbeing [27,28,29,30].

## 4. Discussion

Both the societal burden of burn injuries, as well as those of individual burn patients, are well characterized in the literature [35,36]. The concept of ‘disability’ has been widely used to help measure this burden, and the DALY enjoys widespread use in global health. However, this scoping review of six databases was unable to find a single study directly soliciting DWs—a crucial component of the DALY formula—from burn patients. Moreover, although burn patients are frequently surveyed to assess various physical and psychosocial function after injuries [37,38], investigation regarding their perspectives on the dominant philosophical models of disability do not appear to exist. This matters because, as we have discussed above, these assumptions determine what does and does not count as a disability, which in turn gives patients weaker or stronger claims to healthcare resources. Our scoping review highlights a significant gap in burn literature that must be addressed to provide better insight regarding the needs of burn-injured people and to guide resource allocation.

Of the three dominant philosophical models, we hypothesize burn patient descriptions of their experience with disability appear to most closely aligned with the ‘welfare model’ of disability. This model identifies the intrinsic harm of a disability with a reduction in well-being that is the result of a disability, rather than the disability itself [18,20]. The disability is therefore instrumentally harmful—i.e., a hand burn contracture would be more disabling in a context where most people are manual laborers, compared to a context where other occupations are more available. Supporting this hypothesis are some direct patient quotes from the literature included in our studies, such as ‘I was expecting to change my job very soon... but I lost everything, I lost the chance to change my job after the accident...’ [27] Naturally, the context of a disability is significant within this framework [20]. Therefore, patients with manual occupations report significant burdens of disease and cite resumption of work as a significant factor to social adjustment and burn recovery [27,28].

This theory explains the importance of environments in shaping burn patient perspectives of injury and disability [30]. Especially in low resource communities in a global health context, the concept of instrumentally harmful burn injuries are associated with employment and even psychosocial aspects such as self- image. For example, a participant in Rossi et al. reported, ‘I used to work at home, fixing everything... Now I can’t do it anymore... I feel very depressed’ [28]. These results corroborate, for example, investigations in South Africa that show one fifth of families report a decline in food consumption after burn injuries, which was problematic because it limited return to work and income [39]. Furthermore, a majority of participants in Ciofi-Silva et al. reported changing how they dressed to limit scar exposure to sunlight or other individuals in their environments [27]. However, due to this, these patients reported significant difficulties in participating in outdoor social activities, described as important aspects of their community integration For example, the reduction of well-being because of disability is conveyed in this quote from a patient interview, ‘Why do you have this mark? Why don’t you care for yourself and see a plastic surgeon? You will never find a boyfriend with this arm! [27]. Therefore, assessing disability and burden of disease following burn injury should account for the context and environment.

Literature regarding patient perspectives on the philosophy of disability is similarly limited in other fields. However, some studies exist supporting the ‘welfare model.’ For example, one study interviewed women after breast cancer treatment to provide insight into how women view disabilities related to work. Their results suggest the disability suffered as a result of breast cancer was tied to larger concerns of overall reductions in welfare such as ‘less understanding employers’ or ‘lacking the advantages of a structured life’ [40]. Additionally, patients suffering traumatic brain injury (TBI) focused on disability related to mobility and employment, whereas caregivers focused on self-care as important to patients [41]. These results again suggest the link between disability and its impact on the reduction of well-being, as is described by the ‘welfare model’ [20].

In terms of general patient perspectives, the data presented in the four articles that met inclusion criteria are limited in their generalizability. However, this scoping review only identified one study that used the ICF in burn research, potentially due to the lack of consensus. Furthermore, although comprehensive measurement of injury and disease outcomes exist in burn literature, these measurements do not capture patient insight regarding weights of burn sequalae contributing to disability [42,43]. For example, the ICF seeks to better understand the impact of burn injury and disability on patients by considering psychosocial and environmental factors, but does not provide insight into patient perspectives regarding their disabilities. Several scales exist, such as the Patient Reported Outcomes Measurement Information System (PROMIS) or Burn Specific Health Scale (BSHS), that capture patient reported symptom measures [44,45]. However, to date, no quantitative scales designed to assess patient perspectives on disabilities after burn injury exist [46].

The difficulty attributable to this lack of consensus is due to the multifactorial nature of DW interpretations and the lack of qualitative research that utilizes philosophical theory. Based on the results of our scoping review, we suggest a more ‘ad fontes’ approach to research regarding patient-reported burn outcomes. This would entail close examination of the philosophical foundations of concepts used by burn-injured people and healthcare providers when discussing concepts such as “disability”. This could be achieved by closer collaboration with bioethicists. For example, if empirical research suggests—as our study does, though with admittedly few data points—that most burn-injured people’s perspectives align closely with the ‘welfare model’ of disability, then this implies burn injuries are instrumentally harmful and reductions in wellbeing should be the major focus. Future studies should investigate modifiable social, biological, or psychological factors that improve well-being. This is a subtle but paradigmatic shift away from a focus on being ‘burn-less’, and towards what truly matters: human well-being. Currently, the Burn Model System, a federally funded, multi-center program, is dedicated to research on long-term burn recovery by collecting patient-reported outcomes and providing recommendations to improve care [47]. Future research may want to utilize qualitative interviews grounded in philosophical models of disability to elicit patient perspectives on appropriate DWs in burn injuries.

Some limitations of this study include potential selection bias from the search terms and choices of database for study collection. Furthermore, strict inclusion criteria and our focus on studies that explicitly investigated burn patient perspectives on disability and DWs may have excluded studies investigating similar themes. Strengths of this scoping review include the use of philosophy and psychology databases as well as a database for allied health partners. Additional strengths include the updated nature of the study and its pre-registration, the specificity of the research questions, and the multi-disciplinary perspectives of this study.

## 5. Conclusions

This scoping review highlights a significant gap in the literature, with no studies directly investigating burn patient perspectives on burn DWs. Current DWs are derived from public and health expert opinions with limited input from patients, despite the DALY’s goal of measuring the ’individual burden of disease.’ Further research should investigate DWs from burn patients’ perspectives. Second, the limited primary participant data extrapolated from this review suggest burn-injured people view their injuries as instrumentally detrimental, which aligns most closely with the welfarist view of disability. More definitive investigations into determining which philosophical model of disability best aligns with burn patient experiences are necessary to ground the health economics of burns in sound theory.

## Figures and Tables

**Table 1:** Disability weights for burn conditions from the Global Burden of Disease studies from 2004 to 2019.

Health State *	GBD 2019	GBD 2010	GBD 2004
Burns of <20% TBSA without lower airway burns: short term, with or without treatment.	0.141	0.096	0.157
Burns of <20% TBSA or <10% TBSA if head or neck, or hands or wrist involved: long term, with or without treatment.	0.016	0.018	0.002
Burn of ≥20% TBSA: short term, with or without treatment.	0.314	0.333	0.455
Burn of ≥20% TBSA or ≥10% TBSA if head or neck, or hands or wrist involved: long term, with treatment.	0.135	0.127	0.255
Burns of ≥20% TBSA or 10% TBSA if head or neck, or hands or wrist involved: long term, without treatment.	0.455	0.438	0.255

* Adapted from: WHO methods and data sources for global burden of disease estimates 2000–2019. [1] % TBSA: Percent Total Body Surface Area.

**Table 2:** Summary of philosophical models of disability.

Proposed Definitions	Medical	Social	Welfarist	ICIDH-1 Model	Nagi Model	Verbrugge and Jette	IOM-1 and IOM-2 Model
	A measure that deviates from the scientific or biological truths for a species [20].	Limit or loss of opportunities to take part in community life because of physical and social barriers [21].	Disability is a harmful state resulting from interactions between a person’s biology and psychology and surrounding environment [22].	In the context of health experience, any restriction or lack of ability to perform an activity in the manner or within the range considered normal for a human being [21].	Pattern of behavior that evolves in situations of long-term or continued impairments that are associated with functional limitations [21].	Disability is experiencing difficulty doing activities in any domain of life due to a health or physical problem [22].	The expression of a physical or mental limitation in a social context—the gap between a person’s capabilities and the demands of the environment [21].

**Table 3:** Literature search database composition.

Database	Total Number of Results
PubMed	197
Embase	168
Cumulated Index to Nursing and Allied Health Literature (CINAHL)	68
Web of Science	145
Psycinfo	186
PhilPaper	0

**Table 4:** Included article descriptions.

Author	Intervention(s)	Patient *n*	Demographics	Assessment Tools	Outcomes Measured
Ciofi-Silva et al., 2010 [27]	Semi-structured interview	44	Sao Paulo, Brazil	Not Available	Work, leisure, relationships, religious ties, educational activities, habits
Dunpath et al., 2014 [30]	Semi-structured interview	5	Durban, South Africa	International Classification of Function, Disability and Health framework to assess responses to seven open ended questions.	Burn experience, physiotherapy, pain experience, future outlook on life
Rossi et al., 2009 [28]	Direct observation and semi-structured interviews	19	Sao Paulo, Brazil	Not Available	Resuming work and functional ability, meaning of quality of life: having autonomy, body image, having leisure, interpersonal relationships
Russell et al., 2013 [29]	Semi-structured interview and self-report psychological assessment	82	Texas, TX, USA	Tennessee Self Concept Scale, 2nd edition, Young Adult Self Report, and Structured Clinical Interview for DSM-IV Axis I disorders.	Physical function, appearance, sexuality, moral conduct, personal values, academics and work, identity
